# The prevalence of trimester-specific dietary supplements and associated factors during pregnancy: An observational study

**DOI:** 10.3389/fphar.2023.1135736

**Published:** 2023-04-06

**Authors:** Ying Liu, Nafei Guo, Haoxue Feng, Hui Jiang

**Affiliations:** Shanghai First Maternity and Infant Hospital, Shanghai, China

**Keywords:** dietary supplements, nutrition, folic acid, iron, calcium, pregnant women

## Abstract

**Objectives:** This study aimed to assess the prevalence of trimester-specific dietary supplements (DS)s use and their possible correlates during pregnancy.

**Method:** Pregnant women were convenience sampled and recruited from a comprehensive obstetric care center in Shanghai, China. Data relating to the use of DS during pregnancy, social support and other social-demographic and obstetric data were collected. Trimester-specific DS use and factors related DS were explored.

**Results:** Of the 2803 women participating in this study, 94.8%, 96.2%, 93.8%, and 94.4% reported the use of at least one DS during pregnancy (all trimesters) and in the first, second, and third trimesters of pregnancy, respectively. Significant differences were noted in the use of DS containing folic acid, calcium, iron, vitamins, and docosahexaenoic acid (DHA), during the three trimesters of pregnancy. A higher proportion of DS use was negatively associated with certain categories of pregnant woman, including unemployed/housewife, low education level, obese, and low social support. A positive association was identified with gestational age.

**Conclusion for practice:** Considering the high prevalence of DS use during pregnancy, future studies are needed to evaluate the accuracy and suitability of DS usage during pregnancy.

## Introduction

Maternal nutritional status during pregnancy is essential for both maternal and fetal health. Of particular importance are micronutrients such as folate acid, iron, calcium, and zinc, all of which are considered to be positively related to fetal brain development (Wu et al., 2012; Parisi et al., 2019). However, it has been suggested that diet alone may not fully meet the heightened nutritional requirements during pregnancy (Wu et al., 2012; Mousa et al., 2019). Furthermore, micronutrient deficiencies are common during pregnancy, especially in resource-poor environments and in low- and middle-income countries (Keats et al., 2019; Lander et al., 2019; Parisi et al., 2019). Dietary supplements (DS)s, as defined by the Food and Drug Administration (FDA), are non-drug products that contain dietary ingredients such as vitamins, minerals, and amino acids, that can be used to supplement the diet. The use of DS is a common practice in pregnant women worldwide and aims to address nutritional gaps in their diets of pregnant women so that they may achieve beneficial outcomes in both fetal and maternal health. Among these, the use of folic acid supplements during pregnancy has been recommended by the World Health Organization (WHO), China, and many other countries to prevent neural tube defects (NTDs) (Ren, 2015; Chitayat et al., 2016). Calcium supplements are also used to prevent pregnancy-related hypertensive disorders (Omotayo et al., 2016; Mousa et al., 2019).

Variations on the prevalence of DS use during pregnancy are evident across different countries. The reported prevalence of at least one DS use during pregnancy was 75.3% in Japan (the majority of participants were in mid-to-late pregnancy) (Sato et al., 2013), 77.6% in the United States (Branum et al., 2013; Jun et al., 2020), 78.0% in Sweden (the first trimester) (Bärebring et al., 2018), 81.4% in Norway (the first 4–5 months of pregnancy) (Haugen et al., 2008), 85% in Finland (Arkkola et al., 2006), and 91.0% in Brazil (Miranda et al., 2019). Epidemiological data on the use of DS by Chinese pregnant women is sparse. One study in the Sichuan Province of China reported the use of DS in 81.8% of pregnancies although this data was collected retrospectively (after delivery and before the women had been discharged from the hospital) (Tang et al., 2017). Another study, carried out in Taiwan, investigated the use of DS during pregnancy among 366 women who underwent assisted reproductive technology (Lin et al., 2019).

Furthermore, the use of DS has been reported to relate to a variety of factors at both the individual and national levels. Studies have shown that socioeconomic status, such as age, gender, educational level, employment status, income, marital status, residence, and medical insurance status, are all factors that could affect the use of DS in a general population (Kofoed et al., 2015; Mohd Ashri et al., 2021; Mohsen et al., 2021; Rontogianni et al., 2021). However, how sociodemographic and obstetric-specific factors contribute to the use of DS during pregnancy largely remains unclear. Some previous studies investigated factors related to the use of DS during pregnancy (Bärebring et al., 2018; Jun et al., 2020). One study in Sweden showed that gestational age, parity, birthplace, income, education, and employment, were all factors related to the use of DS in the first trimester (Bärebring et al., 2018). Young age, low family support, and early pregnancy were reported to relate to lower levels of DS use in pregnant women in the United States (Jun et al., 2020). Evidence is emerging in the literature to indicate there are socioeconomic disparities in the use of DS in the general population (Mohd Ashri et al., 2021; Mohsen et al., 2021; Rontogianni et al., 2021). Disparities in the use of DS during pregnancy may have a negative impact on the health of mothers and their offspring; these factors need to be investigated urgently.

It is vital that we understand the specific use of DS during pregnancy, consider important implications for the formulation of appropriate and effective interventions for the appropriate use of DS, and produce public health policies relating to maternal nutrition in pregnancy. Under this perspective, the objectives of the present study were to: (a) measure the prevalence of the trimester-specific use of DS, and (b) investigate the factors related to the use of DS by women. We hypothesized that the prevalence of DS use will vary across the different periods of pregnancy and that the use of DS might be related to sociodemographic and obstetric characteristics in pregnant women.

## Materials and methods

This was a cross-sectional survey that was conducted to investigate the trimester-specific use of DS by pregnant women in Shanghai, China. We also investigated the factors associated with the use of DS. The paper was written in accordance with the Strengthening the Reporting of Observational Studies in Epidemiology guidelines.

### Study population

A convenience sampling method was used to recruit pregnant women who registered their pregnancy in the study hospital and attended their routine antenatal visits. Given the utilization of multivariate analysis in the present study, a sample size of 150–300 participants is recommended to ensure a stable test, with 10–20 participants per independent variable being deemed appropriate, and given the estimated number of independent variables that potentially relate to the use of DS in this study, which is 15. And from epidemiological point of view, considering that 300,000 deliveries are reported in the study setting each year, we selected a 10% (n = 3000) sample for our survey. The inclusion criteria were (1) ≥ 18 years of age, (2) pregnancy confirmed by B ultrasound, (3) competent in Mandarin, (4) able to use a mobile phone, and (5) agreed to participate in the study. The exclusion criteria were as follows: (1) cognitive or mental disorders and (2) severe pregnancy complications. Of the 3000 women approached, 124 refused to participate, while 2803 completed the questionnaires (a response rate of 93.4%). This study was reviewed and approved by the Ethics Committee of the Shanghai First Maternity and Infant Hospital (Reference: KS19101).

### Measures

#### DS use

To the best of our knowledge, there are no widely recognized tools available to investigate the use of DS by women during pregnancy. Referring to a series of related studies (Tang et al., 2017; Lin et al., 2019; Miranda et al., 2019), we developed a self-reported checklist to assess the current use of DS by women over the previous 4 weeks of pregnancy. The checklist included seven common DS items: folic acid, calcium, iron, iodine, zinc, vitamins, and DHA. Vitamins included both multi-vitamins and single vitamins such as vitamins A, C, D, E, *etc.*, but ruled out folic acid supplementation alone. Although folic acid is also a vitamin, we have listed this agent separately because it plays a vital role during pregnancy. We also provide an “other” response for women to record the brand name of the DS they used if they did not know the ingredients. The “other” response was categorized into seven items by the researcher based on ingredients. A panel of experts (three experts in maternal nutrition and three researchers) examined the DS use checklist for validity. A pilot survey of 40 pregnant women showed that the test-retest reliability over 2 weeks of all DS items was 0.75–0.86.

#### Social-demographic data

Social-demographic characteristics included age, ethnicity (Han Chinese or ethnic minority), educational level (primary, secondary, college, Bachelor or Master and above), occupation (employed or unemployed/housewife), marital status (married or single/divorced), local residence (Shanghai or non-Shanghai), medical insurance (with medical insurance or no medical insurance), and social support. Except for social support, all social-demographic characteristics were extracted from electronic medical records. Social support was evaluated by the social supported rating scale. This scale was designed by Xiao in 1986 and was revised in 1994 (Xiao, 1994). This is a comprehensive scale that evaluated the level of objective support, subjective support, and use of support received by participants. The sum of scores ranged from 12 to 66; a higher score indicated a higher level of social support. This scale is widely used in the Chinese population with good reliability and validity; previous research showed that Cronbach’s α coefficient was 0.89 and test-retest reliability was 0.92 (Xiao, 1994). In the current study, Cronbach’s α coefficient was 0.87.

#### Obstetric data

We considered a range of obstetric characteristics, including parity (primipara or multipara), mode of conception (natural conception or artificial conception), gestational age, pregnancy risk grading (green, yellow, or orange), pre-pregnancy body mass index (PPBMI), and history of adverse pregnancy. The pregnancy risk grading was routinely evaluated for all Chinese pregnant women who registered their pregnancy in a hospital l. The severity of pregnancy risk was classified into five colors: green (low risk), yellow (general risk), orange (high risk), red (very high risk) and purple (infectious disease). PPBMI was categorized into four groups according to the WHO recommendation on adult body mass index (BMI) classification for the Asian population: underweight (BMI <18.5 kg/m^2^); normal weight (18.5 kg/m^2^ ≤ BMI <24.0 kg/m^2^); overweight (24.0 kg/m^2^ ≤ BMI <28.0 kg/m^2^); and obese (BMI ≥28.0 kg/m^2^) (Consultation, 2004). Histories of abnormal pregnancy included miscarriage (≥3 times), a history of preterm birth, perinatal death, birth defects, a history of ectopic pregnancy, a history of trophoblastic disease, a history of previous pregnancy complications, and comorbidities.

#### Data collection

This study was conducted over 1 year (May 2020–May 2021). The study involved a paper-free survey; this was due to the forced restriction of unnecessary contact during the COVID-19 pandemic. The research staff approached pregnant women who were waiting for their routine antenatal visits and explained the basic details of the study to eligible participants. After informed consent was obtained, a quick response code (QR code) for the electronic questionnaire was presented. This allowed the study participants to scan the QR code using their smartphones to complete the survey online. To ensure the uniqueness of each participant, each pregnant woman’s medical card number would only be allowed to participate once in this study. The questionnaire was completed in approximately 3–5 min during pilot testing. The research staff were nearby to provide assistance with the questionnaire if necessary; these staff allowed an adequate time for the patients to rest and ask questions.

#### Statistical analyses

Data were analyzed using the Statistical Package for Social Sciences version 20.0 for Windows (IBM Company, Chicago, United States). Social-demographic characteristics, obstetric characteristics, and the use of DS during pregnancy, were reported using descriptive statistics. Trimester-specific DS use was tested by the Chi-squared test and advanced pairwise comparisons were adjusted by the Bonferroni correction. Group differences in the overall use of DS were tested by t-tests or by one-way analysis of variance. And chi-square tests were used to analyze group differences in each DS based on sociodemographic and obstetric data. Multiple linear regression analysis was also performed to explore factors related to the use of DS. Social-demographic and obstetric characteristics that were related to DS use in univariate analysis (*p* < 0.05) were then re-evaluated by multiple regression analysis with a stepwise procedure (entry 0.05, removal 0.10). For all analyses, the alpha level of significance was set to 0.05.

## Results

### Participant characteristics

Tables 1, 2 show the social-demographic and obstetric characteristics of the participants along with the various aspects of DS use in each group. The age of the participating women ranged from 18 to 45 (mean: 30.28 ± 3.81 years), and the mean gestational age was 22.35 ± 4.49 weeks. Most of the participants were Han Chinese (98.0%), employed (83.8%), held a bachelor’s degree or above (62.6%), and had medical insurance (87.5%).

**TABLE 1:** Relationships in the use of different types of dietary supplements with social-demographic characteristics in pregnant women (N = 2803).

Variables	n (%)	No. of types mean ± SD	t/F value	*p*-value
Age			1.789	0.074
<35 years	2343 (83.6)	2.139 ± 1.239		
≥35 years	460 (16.4)	2.252 ± 1.236		
Ethnicity			0.865	0.387
Han Chinese	2746 (98.0)	2.155 ± 1.239		
Ethnic minority	57 (2.0)	2.298 ± 1.239		
Employment^a^			6.280	**<0.001**
Employed	2342 (83.8)	2.217 ± 1.236		
Unemployed/housewife	453 (16.2)	1.821 ± 1.194		
Education level^a^			22.403	**<0.001**
Primary education	145 (5.2)	1.510 ± 0.980		
Secondary education	234 (8.4)	1.957 ± 1.130		
College	665 (23.8)	2.012 ± 1.205		
Bachelor	1299 (46.5)	2.222 ± 1.218		
Master’s and above	451 (16.1)	2.468 ± 1.347		
Marital status			2.349	**0.019**
Single/divorced	127 (4.5)	1.906 ± 1.312		
Married	2676 (95.5)	2.170 ± 1.235		
Local residence			2.931	**0.003**
Shanghai	843 (30.1)	2.262 ± 1.280		
Non-Shanghai	1960 (69.9)	2.113 ± 1.219		
Medical insurance			5.807	**<0.001**
No medical insurance	351 (12.5)	1.801 ± 1.142		
Have medical insurance	2452 (87.5)	2.209 ± 1.244		
Social support			5.773	**0.003**
Low	1036 (37.0)	2.085 ± 1.218		
Medium	901 (32.1)	2.131 ± 1.234		
High	866 (30.9)	2.273 ± 1.263		

^a^ missing data.

Bold values indicate statistical significance.

**TABLE 2:** Relationships in the use of different types of dietary supplements with women’s obstetric characteristics (N = 2803).

Variables	n (%)	No. of types mean ± SD	t/F value	*p*-value
Gestational age			148.591	**<0.001**
First trimester	915 (32.6)	1.644 ± 0.982		
Second trimester	982 (35.0)	2.244 ± 1.216		
Third trimester	906 (32.3)	2.583 ± 1.311		
PPBMI			3.102	**0.026**
Underweight	320 (11.4)	2.163 ± 1.317		
Normal weight	2014 (71.9)	2.174 ± 1.228		
Overweight	398 (14.2)	2.151 ± 1.269		
Obese	71 (2.5)	1.718 ± 0.944		
Conception method			0.847	0.397
Natural conception	2581 (92.1)	2.164 ± 1.246		
Artificial conception	222 (7.9)	2.090 ± 1.162		
Parity			1.065	0.287
Primipara	2118 (75.6)	2.172 ± 1.255		
Multipara	685 (24.4)	2.114 ± 1.189		
History of adverse pregnancy			0.903	0.366
No	2465 (87.9)	2.166 ± 1.244		
Yes	338 (12.1)	2.101 ± 1.202		
Embryo number			0.085	0.993
Singleton	2754 (98.3)	2.158 ± 1.238		
Twin	49 (1.7)	2.143 ± 1.307		
Pregnancy risk grading			0.202	0.817
Green	1173 (41.8)	2.148 ± 1.239		
Yellow	1465 (52.3)	2.170 ± 1.243		
Orange	165 (5.9)	2.115 ± 1.207		

PPBMI: pre-pregnancy body mass index.

Bold values indicate statistical significance.

The prevalence of DS use during pregnancy (all trimesters) and in the first, second, and third trimester of pregnancy was 94.8%, 96.2%, 93.8%, and 94.4%, respectively. 278 (9.9%) of the women chose an “other” option. The “other” responses mainly include some brand names of DS which could be categorized into folic acid, multi-vitamins, and DHA. But also include some traditional Chinese medicine, such as bird’s-nest, donkey-hide gelatin, *etc.* Our data showed that the mean number of different types of DS used was 2.16 ± 1.24.

### Trimester-specific use of DS

Table 3 shows the seven types of DS use during pregnancy (all trimesters) and in the first (n = 915), second (n = 982) and third trimester (n = 906) of pregnancy. Significant differences were noted in the use of folic acid, calcium, iron, vitamins, and DHA supplements (all *p* < 0.001) during the three trimesters, as determined by the Chi-squired test. Pairwise comparisons further showed that the use of folic acid supplements decreased as the pregnancy progressed. Peak use (93.1%) was noted in the first trimester; this rate decreased dramatically (by 49.4%) in the second trimester and reduced even further during the third trimester. In contrast, the use of iron supplements increased as the pregnancy progressed; the use of these supplements was. relatively low (7.9%) in the first trimester but then increased during the second trimester and reached a peak (61.7%) during the third trimester. The use of calcium, vitamins, and DHA supplements, all increased during the second trimester and remained stable in the third trimester. The use of iodine and zinc supplements was extremely low throughout pregnancy (all rates <5.0%).

**TABLE 3:** Trimester-specific dietary supplement use (N = 2803).

Type of dietary supplements	All trimester n = 2803 n (%)	First trimester n = 915 n (%)	Second trimester n = 982 n (%)	Third trimester n = 906 n (%)	*p*-value
Folic acid use	1596 (56.9)	852 (93.1)^a^	429 (43.7)^b^	315 (34.8)^c^	**<0.001**
Calcium use	1636 (58.4)	159 (17.4)^a^	749 (76.3)^b^	728 (80.4)^b^	**<0.001**
Iron use	863 (30.8)	72 (7.9)^a^	232 (23.6)^b^	559 (61.7)^c^	**<0.001**
Iodine use	41 (1.5)	11 (1.2)^a^	15 (1.5)^a^	15 (1.7)^a^	0.707
Zinc use	87 (3.1)	18 (2.0)^a^	37 (3.8)^a^	32 (3.5)^a^	0.052
Vitamins use	1078 (38.5)	283 (30.9)^a^	399 (40.6)^b^	396 (43.7)^b^	**<0.001**
DHA use	747 (26.7)	109 (11.9)^a^	343 (34.9)^b^	295 (32.6)^b^	**<0.001**

DHA: docosahexaenoic acid. a, b, c Each subscript letter denotes a subset of trimester categories whose column proportions do not differ significantly from each other at the 0.05 level.

Bold values indicate statistical significance.

### Factors related to the number of types DS use

Univariate analysis showed that employment, educational level, marital status, local residence, medical insurance, social support, gestational age, and PPBMI, were all factors that were significantly related to the number of DS types used by pregnant women (Table 1; Table 2).

Multiple linear regression analysis showed that the use of a higher number of DS types was negatively associated with several factors: unemployed/housewife (B = −0.180, *p* = 0.010), low educational level (primary education: B = −0.459, *p* < 0.001; college: B = −0.158, *p* = 0.001), obese (B = −0.372, *p* = 0.008) and low social support (B = −0.168, *p* = 0.002), but was positively associated with gestational age (second trimester: B = 0.611, *p* < 0.001; third trimester: B = 0.907, *p* < 0.001) (Table 4). Factors related to folic acid, calcium, iron, iodine, zinc, vitamins, and DHA use separately were presented in the Supplementary File.

**TABLE 4:** Multivariable analysis of factors related to the number of different dietary supplements used by pregnant women.

Variables	B	SE	β	t	*p*-value*
Constant	1.889	0.143		13.231	**<0.001**
Employment (Ref: Employed)					
Unemployed/housewife	−0.180	0.069	−0.054	−2.591	**0.010**
Education level (Ref: Bachelor)					
Primary education	−0.459	0.110	−0.082	−4.183	**<0.001**
Secondary education	−0.147	0.085	−0.033	−1.721	0.085
College	−0.185	0.056	−0.064	−3.321	**0.001**
Master’s and above	0.232	0.065	0.069	3.580	**<0.001**
Gestational age (Ref: First trimester)					
Second trimester	0.611	0.053	0.236	11.473	**<0.001**
Third trimester	0.907	0.054	0.343	16.680	**<0.001**
PPBMI (Ref: Normal weight)					
Underweight	−0.028	0.070	−0.007	−0.407	0.684
Overweight	0.033	0.064	0.009	0.513	0.608
Obese	−0.372	0.141	−0.047	−2.649	**0.008**
Social support (Ref: High level)					
Low level	−0.168	0.054	−0.065	−3.116	**0.002**
Medium level	−0.101	0.055	−0.038	−1.830	0.067

PPBMI, refers to pre-pregnancy body mass index. *Marital status, localresidence, and medical insurance did not statistically affect number of types of dietary supplements use (*p* > 0.05).

Bold values indicate statistical significance.

## Discussion

In the present study, we investigated the use of DS by women during pregnancy as well as the social-demographic and obstetric factors related to the use of DS. This is the first contemporary study to describe the trimester-specific prevalence of DS use in a population of Chinese pregnant women. In this study, the utilization rate of DS among pregnant women in Shanghai, China, was much higher than that reported in other counties (Arkkola et al., 2006; Haugen et al., 2008; Branum et al., 2013; Sato et al., 2013; Miranda et al., 2019), thus suggesting a crucial need for healthcare providers to discuss DS use and guide the use of DS during pregnancy.

We also identified a relatively higher proportion of women taking folic acid supplements during pregnancy (93.1%, 43.7%, and 34.8%, during the first second, and third trimesters, respectively) when compared to the study of a Swedish cohort (74.4% used folic acid in the first trimester compared to 43.2% in the third trimester). However, our results were similar to those reported by a previous study conducted in four cities in China, which found that 95.1% of pregnant women took folic acid supplements during pregnancy or 3 months prior to pregnancy (Cui et al., 2021). The reported high use of folic acid supplements reflects the fact that folic acid supplementation is common in China. The typical Chinese main staple (rice) is not fortified with folate acid, thus resulting in a high prevalence of NTDs in China. To reduce the high prevalence of NTDs, the Chinese government started a nation-wide program in 2009 that provides all women with rural household registration who plan to become pregnant with free folic acid supplements (Ren, 2015).

But it is indicated that socioeconomic disparities in the utilization of folic acid during pregnancy still exist (Liu et al., 2017). Furthermore, routine empirical folic acid supplementation of 0.4 mg/day is recommended by the Chinese Nutrition Society from 3 months prior to conception to the first trimester of pregnancy, in addition to the consumption of folate-rich foods (Wang et al., 2016; Yang et al., 2018). The overall prevalence of folic acid supplement use was 75.62% when reported by a National Free Preconception Health Examination Project of 907,720 rural Chinese women (Liu et al., 2017). The periconceptional folic acid supplementation program has successfully reduced the prevalence of NTDs in China (Cui et al., 2021).

In addition, the demand for additional calcium during pregnancy has been well-established (Hacker et al., 2012). An adequate intake of calcium is especially critical during pregnancy because of its potential effect on maternal and fetal bone health (Hacker et al., 2012). Furthermore, calcium may also reduce the risk of gestational diabetes mellitus and pregnancy-related hypertensive disorders (Khaing et al., 2017; Osorio-Yáñez et al., 2017; Hofmeyr et al., 2018). A previous study in China showed that calcium supplementation was significantly associated with a lower preterm birth risk (Liu D. et al., 2021). Routine calcium supplementation is strong recommended by the WHO to prevent preeclampsia (Omotayo et al., 2016). Furthermore, it has been frequently reported that women do not consume the recommended quantity of calcium from their routine diets, even in developed countries (Hacker et al., 2012). It has been reported that annual milk consumption in China is the lowest in the world and that 90% of the Chinese population is at risk of an inadequate calcium intake (He et al., 2016). The dietary reference intake for calcium among pregnant women in China is 800–1,000 mg/day (Liu X. H. et al., 2021). The typical Chinese diet does not usually meet the increased calcium requirements during pregnancy (Wang et al., 2010; Liu D. et al., 2021). A high prevalence of calcium deficiency was previously reported in pregnant women in Chengdu, China (Wang et al., 2010). These facts may explain the high rate (>75% in the second and third trimesters of pregnancy) of calcium supplement use in the current study.

Along with folate acid and calcium, iron is one of the nutrients for which dietary intake alone cannot realistically meet the increased demands of pregnancy. In addition, due to the low bioavailability of dietary iron, iron supplementation is usually recommended for women during pregnancy to prevent and treat maternal iron deficiency anemia (IDA), but must be used with caution (Hacker et al., 2012; Siu, 2015). In fact, iron requirements fluctuate across pregnancy. In the first trimester, iron requirements decrease; this is due to the cessation of menses and the improved absorption capacity of iron from the diet during pregnancy (EFSA Panel on Dietetic Products and Allergies, 2015). In the second trimester, due to expansion of plasma volume and red cell mass, iron requirements increase; they also continue to increase during the third trimester as iron accumulates in the placenta (Hacker et al., 2012). A prospective multi-racial study reported that elevated iron stores in the first trimester were positively associated with the development of gestational diabetes (Rawal et al., 2017). Actually, routine iron supplementation for all women is not recommended (Pavord et al., 2012). And given that iron requirements vary during pregnancy and the adverse effects of both iron deficiency and overload (Fu et al., 2016; Rawal et al., 2017), precise recommendations exist for iron intake within the context of body iron status, pregnancy stage, and dietary iron intake. In China, the estimated prevalence of anemia is 23.5% (entire pregnancy), and 2.7%, 14.7%, and 16.6% in the first, second, and third trimesters, respectively, as defined by a hemoglobin concentration of 110 g/L at any stage of pregnancy (Lin et al., 2018). To achieve the goal set in The National Nutrition Plan (2017–2030), which was issued by the General Office of the State Council, P.R. China, to reduce the prevalence of anemia in pregnant women to <10% by 2030 (Council, 2017), there are still many measures that require attention. In the hospital where the study was conducted, 100–200 mg/day of elemental iron is usually recommended for women with IDA (Association, 2014). Of the pregnant women in the current study, 7.9%, 23.6%, and 61.7%, took iron supplements in the first, second, and third trimesters, respectively. This result is consistent with the increased iron requirement during mid-late pregnancy (Hacker et al., 2012). Similarly, an Australian cohort study reported the use of triple iron supplementation from the first-to-third trimesters (Livock et al., 2017).

In this study, vitamin supplements were used by 30.9%, 40.6%, and 43.7% of women in the first, second, and third trimesters; these figures are relatively lower than those reported by Savard et al. (86.1%, 84.8%, and 78.5%, respectively) (Savard et al., 2018). This finding can be explained by the fact that prenatal multivitamins are the most favored supplement taken by the Western population (Savard et al., 2018). Vitamin D, an essential fat-soluble vitamin, is very important during pregnancy. It is reported that a considerable proportion (40.7%) of Chinese pregnant women suffer from vitamin D deficiency (Yang et al., 2021). In this study, we did not investigate the intake vitamin D separately. However, it is worth mentioning that calcium supplements are almost always supplemented with vitamin D. In this study, calcium supplements were used by 17.4%, 76.3%, and 80.4% of women in the first, second, and third trimesters. In addition, DHA, a key and essential n-3 long-chain polyunsaturated fatty acid, is essential for the fetal central nervous system along with retina function development and immunomodulation (Massari et al., 2020). Dietary DHA can be readily found in seafood, particularly deep-sea fish, such as salmon, herring, and anchovies. In China, women are encouraged to eat deep-sea fish 2–3 times a week during pregnancy (Wang et al., 2016; Yang et al., 2018). Compared to other ethnic groups, the intake of DHA from dietary sources is very low in China (Li et al., 2015; Deng et al., 2017). This finding may explain the higher use of DHA supplements reported in the current study (11.9%–34.9%) than other areas (4.5% in the first trimester and 4.6% in the third trimester in Sweden) (Bärebring et al., 2018). The high proportion of DHA supplement use in Chinese pregnant women may also reflect an increased awareness in the role of DHA in fetal brain development (Li et al., 2015).

This study considered the use of dietary supplements (DS) during pregnancy under both medical guidance and self-administration by pregnant women. Folic acid supplementation during the first trimester is generally a self-initiated practice among expectant mothers for prenatal care. In cases where early prenatal consultations reveal a lack of folic acid intake, physicians may prescribe the supplement accordingly. Clinical assessments are typically used to detect calcium and iron deficiencies, and if such deficiencies are diagnosed, supplementation or prescription may be recommended. However, some pregnant women may choose to take these supplements for preventive purposes. Vitamins and DHA, on the other hand, are typically self-administered by pregnant women.

We also investigated the sociodemographic and obstetric factors related to the use of DS among pregnant women. We found that being unemployed, being a housewife, having a low educational level, low levels of social support, and being obese, were all negatively associated with the number of DS types used by pregnant women. These results are supported by a previous study carried out in Sweden which showed that education level and employment were associated with the use of DS during early pregnancy (Bärebring et al., 2018). A previous study reported that high socioeconomic status was associated with a healthier diet during pregnancy (Poulain et al., 2021). It is possible that socioeconomic status is related to a woman’s ability to purchase DS. The relationship between educational level and DS in the present study was consistent with previous studies conducted in the Sichuan Province of China (Tang et al., 2017) and the United States (Branum et al., 2013). These findings suggest a tendency for women with a higher level of education to make greater efforts to achieve a better nutritional status during pregnancy. This might be explained by the fact that a higher level of education was related to a higher level of health awareness and better health literacy. Social support has been shown to be related to improved adherence to the use of folic acid supplements during pregnancy (Wiradnyani et al., 2016). Our present data suggested that low social support may affect the number of different types of DS used by women during pregnancy. Constant and comprehensive maternal social support is important for healthy dietary habits during pregnancy (Hopkins et al., 2018). Clear and open communication between women and healthcare providers, along with the involvement of family members, may help to improve the use of DS during pregnancy, particularly with regards to the benefits of DS, concerns related to adverse effects, as well as financial barriers (Wiradnyani et al., 2016; Jun et al., 2020). BMI has been reported to be inversely associated with the use of DS in the general population (Rontogianni et al., 2021). Importantly, our data also revealed that obese women are associated with a lower use of DS during pregnancy. As excessive body weight and obesity are known to represent global public health problems in both developed and developing countries, obesity itself has been proven to be associated with adverse health outcomes during pregnancy and over the longer term (Simon et al., 2020). Our research has enhanced our understanding of this field by suggesting that obese women should be supported/educated with regards to their nutritional health during the critical period of pregnancy.

In addition, we observed an increase in the use of DS in married women, those living in Shanghai, and those that had medical insurance. However, these associations did not retain significance in the multivariable model. Although parity was also reported as a factor related to the use of DS during pregnancy in Sweden (Bärebring et al., 2018) and Norway (Haugen et al., 2008), our present data did not concur with these earlier results. This inconsistency may be explained by the different characteristics of the participants in our study. In China, the proportion of primiparous women is much higher within the maternal population (75.6%) than that reported in previous studies carried out in Sweden (42.3%) (Bärebring et al., 2018) or Norway (47.3%) (Haugen et al., 2008).

## Limitations

There are some limitations associated with the present study that we need to consider. First, this study was conducted in one hospital in Shanghai; most participants were highly educated. Second, the use of DS in this study was only evaluated by yes or no questions, the advantage of this instrument is that it provides a time-effective means of providing dietary surveys in a busy clinical setting. A disadvantage, however, is the limited accuracy of the dietary data. Furthermore, we were unable to calculate the actual intake of supplements and food sources. Therefore, it was impossible to compare the actual intake with dietary recommendations in the current study. Future studies should involve a larger and more representative sample and include more detailed dietary investigations. In addition, the use of DS prior to conceptional was not investigated in this study. Finally, the cross-sectional study design precludes the investigation of any specific causal relationships.

## Conclusion

In summary, we demonstrated a high prevalence of DS use in women during pregnancy and the use of DS was associated with employment status, educational level, perceived social support, PPBMI, and gestational age. The findings of the current study emphasized the crucial need for future studies to evaluate the accuracy and suitability of DS usage during pregnancy. Due to the high prevalence of DS use during pregnancy, synergistic efforts between policymakers, maternal and child nutrition experts, and obstetric health workers, are highly recommended to guide the appropriate use of DS during pregnancy, such as education, monitoring, and taking necessary actions.

## Data Availability

The raw data supporting the conclusion of this article will be made available by the authors, without undue reservation.

## References

[B1] Arkkola T. Uusitalo U. Pietikäinen M. Metsälä J. Kronberg-Kippilä C. Erkkola M. (2006). Dietary intake and use of dietary supplements in relation to demographic variables among pregnant Finnish women. Br. J. Nutr. 96 (5), 913–920. 10.1017/bjn20061929 17092382

[B2] Association P. M. B. o. C. M. (2014). Guidelines for the diagnosis and treatment of iron deficiency and iron deficiency anemia in pregnancy. Chin. J. Perinat. Med. (7), 451–454. (In Chinese).

[B3] Bärebring L. Mullally D. Glantz A. Elllis J. Hulthén L. Jagner Å. (2018). Sociodemographic factors associated with dietary supplement use in early pregnancy in a Swedish cohort. Br. J. Nutr. 119 (1), 90–95. 10.1017/S0007114517003270 29198190

[B4] Branum A. M. Bailey R. Singer B. J. (2013). Dietary supplement use and folate status during pregnancy in the United States. J. Nutr. 143 (4), 486–492. 10.3945/jn.112.169987 23365107 PMC4745881

[B5] Chitayat D. Matsui D. Amitai Y. Kennedy D. Vohra S. Rieder M. (2016). Folic acid supplementation for pregnant women and those planning pregnancy: 2015 update. J. Clin. Pharmacol. 56 (2), 170–175. 10.1002/jcph.616 26272218 PMC4738404

[B6] Consultation W. E. (2004). Appropriate body-mass index for Asian populations and its implications for policy and intervention strategies. Lancet 363 (9403), 157–163. 10.1016/S0140-6736(03)15268-3 14726171

[B7] Council G. O. o. t. S. (2017). National nutrition plan (2017-2030). J. Nutr. 39 (04), 315–320. (In Chinese).

[B8] Cui M. Lu X. L. Lyu Y. Y. Wang F. Xie X. L. Cheng X. Y. (2021). Knowledge and intake of folic acid to prevent neural tube defects among pregnant women in urban China: A cross-sectional study. BMC Pregnancy Childbirth 21 (1), 432. 10.1186/s12884-021-03893-4 34154557 PMC8218380

[B9] Deng J. Li X. Ding Z. Wu Y. Chen X. Xie L. (2017). Effect of DHA supplements during pregnancy on the concentration of PUFA in breast milk of Chinese lactating mothers. J. Perinat. Med. 45 (4), 437–441. 10.1515/jpm-2015-0438 27235666

[B10] EFSA Panel on Dietetic Products and Allergies (2015). Scientific opinion on dietary reference values for iron. EFSA J. 13 (10), 4254. 10.2903/j.efsa.2015.4254 PMC13151608 42109813

[B11] Fu S. Li F. Zhou J. Liu Z. (2016). The relationship between body iron status, iron intake and gestational diabetes: A systematic review and meta-analysis. Med. Baltim. 95 (2), e2383. 10.1097/MD.0000000000002383 PMC4718241 26765415

[B12] Hacker A. N. Fung E. B. King J. C. (2012). Role of calcium during pregnancy: Maternal and fetal needs. Nutr. Rev. 70 (7), 397–409. 10.1111/j.1753-4887.2012.00491.x 22747842

[B13] Haugen M. Brantsaeter A. L. Alexander J. Meltzer H. M. (2008). Dietary supplements contribute substantially to the total nutrient intake in pregnant Norwegian women. Ann. Nutr. Metab. 52 (4), 272–280. 10.1159/000146274 18645244 PMC2813797

[B14] He Y. Yang X. Xia J. Zhao L. Yang Y. (2016). Consumption of meat and dairy products in China: A review. Proc. Nutr. Soc. 75 (3), 385–391. 10.1017/S0029665116000641 27334652

[B15] Hofmeyr G. J. Lawrie T. A. Atallah Á N. Torloni M. R. (2018). Calcium supplementation during pregnancy for preventing hypertensive disorders and related problems. Cochrane Database Syst. Rev. 10 (10), Cd001059. 10.1002/14651858.CD001059.pub5 30277579 PMC6517256

[B16] Hopkins A. L. Yeoman M. Ritenbaugh C. (2018). Healthy foods prepared at home: Diet and support as protective strategies during pregnancy for Hispanic women. Ecol. Food Nutr. 57 (2), 140–161. 10.1080/03670244.2018.1423971 29323534

[B17] Jun S. Gahche J. J. Potischman N. Dwyer J. T. Guenther P. M. Sauder K. A. (2020). Dietary supplement use and its micronutrient contribution during pregnancy and lactation in the United States. Obstet. Gynecol. 135 (3), 623–633. 10.1097/AOG.0000000000003657 32028492 PMC7138460

[B18] Keats E. C. Haider B. A. Tam E. Bhutta Z. A. (2019). Multiple-micronutrient supplementation for women during pregnancy. Cochrane Database Syst. Rev. 3 (3), Cd004905. 10.1002/14651858.CD004905.pub6 30873598 PMC6418471

[B19] Khaing W. Vallibhakara S. A. Tantrakul V. Vallibhakara O. Rattanasiri S. McEvoy M. (2017). Calcium and vitamin D supplementation for prevention of preeclampsia: A systematic review and network meta-analysis. Nutrients 9 (10), 1141. 10.3390/nu9101141 29057843 PMC5691757

[B20] Kofoed C. L. Christensen J. Dragsted L. O. Tjønneland A. Roswall N. (2015). Determinants of dietary supplement use-healthy individuals use dietary supplements. Br. J. Nutr. 113 (12), 1993–2000. 10.1017/S0007114515001440 25940747

[B21] Lander R. L. Hambidge K. M. Westcott J. E. Tejeda G. Diba T. S. Mastiholi S. C. (2019). Pregnant women in four low-middle income countries have a high prevalence of inadequate dietary intakes that are improved by dietary diversity. Nutrients 11 (7), 1560. 10.3390/nu11071560 31295916 PMC6682861

[B22] Li Y. Li H. T. Trasande L. Ge H. Yu L. X. Xu G. S. (2015). DHA in pregnant and lactating women from coastland, lakeland, and inland areas of China: Results of a DHA evaluation in women (DEW) study. Nutrients 7 (10), 8723–8732. 10.3390/nu7105428 26506380 PMC4632448

[B23] Lin C. Y. Chen Y. J. Lee S. H. Kuo C. P. Lee M. S. Lee M. C. (2019). Uses of dietary supplements and herbal medicines during pregnancy in women undergoing assisted reproductive technologies- A study of taiwan birth cohort. Taiwan J. Obstet. Gynecol. 58 (1), 77–81. 10.1016/j.tjog.2018.11.015 30638486

[B24] Lin L. Wei Y. Zhu W. Wang C. Su R. Feng H. (2018). Prevalence, risk factors and associated adverse pregnancy outcomes of anaemia in Chinese pregnant women: A multicentre retrospective study. BMC Pregnancy Childbirth 18 (1), 111. 10.1186/s12884-018-1739-8 29685119 PMC5914057

[B25] Liu D. Li S. Lei F. Zhao Y. Cheng Y. Dang S. (2021a). Associations between maternal calcium intake from diet and supplements during pregnancy and the risk of preterm birth in a Chinese population. Eur. J. Clin. Nutr. 75 (1), 141–150. 10.1038/s41430-020-00701-8 32814854

[B26] Liu M. Chen J. Liu J. Zhang S. Wang Q. Shen H. (2017). Socioeconomic inequality in periconceptional folic acid supplementation in China: A census of 0.9 million women in their first trimester of pregnancy. BMC Pregnancy Childbirth 17 (1), 422. 10.1186/s12884-017-1618-8 29246118 PMC5732497

[B27] Liu X. H. Su Y. X. Wang Z. X. Qi H. B. Li T. (2021b). Expert consensus on calcium supplementation for pregnant women in China (2021). J. Pract. Obstetrics Gynecol. 37 (05), 345–347. (In Chinese).

[B28] Livock M. Anderson P. J. Lewis S. Bowden S. Muggli E. Halliday J. (2017). Maternal micronutrient consumption periconceptionally and during pregnancy: A prospective cohort study. Public Health Nutr. 20 (2), 294–304. 10.1017/S1368980016002019 27485466 PMC10262275

[B29] Massari M. Novielli C. Mandò C. Di Francesco S. Della Porta M. Cazzola R. (2020). Multiple micronutrients and docosahexaenoic acid supplementation during pregnancy: A randomized controlled study. Nutrients 12 (8), 2432. 10.3390/nu12082432 32823606 PMC7468952

[B30] Miranda V. I. A. da Silva Dal Pizzol T. Silveira M. P. T. Mengue S. S. da Silveira M. F. Lutz B. H. (2019). The use of folic acid, iron salts and other vitamins by pregnant women in the 2015 pelotas birth cohort: Is there socioeconomic inequality? BMC Public Health 19 (1), 889. 10.1186/s12889-019-7269-0 31277638 PMC6612145

[B31] Mohd Ashri M. H. Abu Saad H. Adznam S. (2021). Socio-demographic characteristics, body weight status and energy intake among users and non-users of dietary supplements among government employees in putrajaya, Malaysia. Nutrients 13 (7), 2248. 10.3390/nu13072248 34210072 PMC8308269

[B32] Mohsen H. Yazbeck N. Al-Jawaldeh A. Bou Chahine N. Hamieh H. Mourad Y. (2021). Knowledge, attitudes, and practices related to dietary supplementation, before and during the COVID-19 pandemic: Findings from a cross-sectional survey in the Lebanese population. Int. J. Environ. Res. Public Health 18 (16), 8856. 10.3390/ijerph18168856 34444605 PMC8395050

[B33] Mousa A. Naqash A. Lim S. (2019). Macronutrient and micronutrient intake during pregnancy: An overview of recent evidence. Nutrients 11 (2), 443. 10.3390/nu11020443 30791647 PMC6413112

[B34] Omotayo M. O. Dickin K. L. O'Brien K. O. Neufeld L. M. De Regil L. M. Stoltzfus R. J. (2016). Calcium supplementation to prevent preeclampsia: Translating guidelines into practice in low-income countries. Adv. Nutr. 7 (2), 275–278. 10.3945/an.115.010736 26980810 PMC4785477

[B35] Osorio-Yáñez C. Qiu C. Gelaye B. Enquobahrie D. A. Williams M. A. (2017). Risk of gestational diabetes mellitus in relation to maternal dietary calcium intake. Public Health Nutr. 20 (6), 1082–1089. 10.1017/S1368980016002974 27964774 PMC5645148

[B36] Parisi F. di Bartolo I. Savasi V. M. Cetin I. (2019). Micronutrient supplementation in pregnancy: Who, what and how much? Obstet. Med. 12 (1), 5–13. 10.1177/1753495X18769213 30891086 PMC6416688

[B37] Pavord S. Myers B. Robinson S. Allard S. Strong J. Oppenheimer C. (2012). UK guidelines on the management of iron deficiency in pregnancy. Br. J. Haematol. 156 (5), 588–600. 10.1111/j.1365-2141.2011.09012.x 22512001

[B38] Poulain T. Spielau U. Vogel M. Dathan-Stumpf A. Körner A. Kiess W. (2021). Changes in diet from pregnancy to one year after birth: A longitudinal study. BMC Pregnancy Childbirth 21 (1), 600. 10.1186/s12884-021-04038-3 34481457 PMC8418026

[B39] Rawal S. Hinkle S. N. Bao W. Zhu Y. Grewal J. Albert P. S. (2017). A longitudinal study of iron status during pregnancy and the risk of gestational diabetes: Findings from a prospective, multiracial cohort. Diabetologia 60 (2), 249–257. 10.1007/s00125-016-4149-3 27830277 PMC6331052

[B40] Ren A. G. (2015). Prevention of neural tube defects with folic acid: The Chinese experience. World J. Clin. Pediatr. 4 (3), 41–44. 10.5409/wjcp.v4.i3.41 26261765 PMC4526837

[B41] Rontogianni M. O. Kanellopoulou A. Markozannes G. Bouras E. Derdemezis C. Doumas M. T. (2021). Prevalence and determinants of sex-specific dietary supplement use in a Greek cohort. Nutrients 13 (8), 2857. 10.3390/nu13082857 34445018 PMC8399686

[B42] Sato Y. Nakanishi T. Chiba T. Yokotani K. Ishinaga K. Takimoto H. (2013). Prevalence of inappropriate dietary supplement use among pregnant women in Japan. Asia Pac J. Clin. Nutr. 22 (1), 83–89. 10.6133/apjcn.2013.22.1.08 23353615

[B43] Savard C. Lemieux S. Weisnagel S. J. Fontaine-Bisson B. Gagnon C. Robitaille J. (2018). Trimester-specific dietary intakes in a sample of French-Canadian pregnant women in comparison with national nutritional guidelines. Nutrients 10 (6), 768. 10.3390/nu10060768 29899222 PMC6024697

[B44] Simon A. Pratt M. Hutton B. Skidmore B. Fakhraei R. Rybak N. (2020). Guidelines for the management of pregnant women with obesity: A systematic review. Obes. Rev. 21 (3), e12972. 10.1111/obr.12972 31943650 PMC7064940

[B45] Siu A. L. (2015). Screening for iron deficiency anemia and iron supplementation in pregnant women to improve maternal health and birth outcomes: U.S. Preventive services task force recommendation statement. Ann. Intern Med. 163 (7), 529–536. 10.7326/M15-1707 26344176

[B46] Tang L. Lee A. H. Yau K. K. W. Hui Y. V. Binns C. W. (2017). Consumption of dietary supplements by Chinese women during pregnancy and postpartum: A prospective cohort study. Matern. Child. Nutr. 13 (4), e12435. 10.1111/mcn.12435 28185404 PMC6866057

[B47] Wang J. Yang F. Mao M. Liu D. H. Yang H. M. Yang S. F. (2010). High prevalence of vitamin D and calcium deficiency among pregnant women and their newborns in Chengdu, China. World J. Pediatr. 6 (3), 265–267. 10.1007/s12519-010-0224-x 20706824

[B48] Wang S. S. Lay S. Yu H. N. Shen S. R. (2016). Dietary guidelines for Chinese residents (2016): Comments and comparisons. J. Zhejiang Univ. Sci. B 17 (9), 649–656. 10.1631/jzus.B1600341 27604857 PMC5018612

[B49] Wiradnyani L. A. Khusun H. Achadi E. L. Ocviyanti D. Shankar A. H. (2016). Role of family support and women's knowledge on pregnancy-related risks in adherence to maternal iron-folic acid supplementation in Indonesia. Public Health Nutr. 19 (15), 2818–2828. 10.1017/S1368980016001002 27181394 PMC10270846

[B50] Wu G. Imhoff-Kunsch B. Girard A. W. (2012). Biological mechanisms for nutritional regulation of maternal health and fetal development. Paediatr. Perinat. Epidemiol. 26 (1), 4–26. 10.1111/j.1365-3016.2012.01291.x 22742599

[B51] Xiao S. Y. (1994). The theoretical basis and research application of "Social Support Rating Scale. J. Clin. Psychiatry, 98–100. (In Chinese).

[B52] Yang C. Jing W. Ge S. Sun W. (2021). Vitamin D status and vitamin D deficiency risk factors among pregnancy of Shanghai in China. BMC Pregnancy Childbirth 21 (1), 431. 10.1186/s12884-021-03889-0 34144704 PMC8214247

[B53] Yang Y. X. Wang X. L. Leong P. M. Zhang H. M. Yang X. G. Kong L. Z. (2018). New Chinese dietary guidelines: Healthy eating patterns and food-based dietary recommendations. Asia Pac J. Clin. Nutr. 27 (4), 908–913. 10.6133/apjcn.072018.03 30045438

