# Supplementary material for: The prevalence of trimester-specific dietary supplements and associated factors during pregnancy: An observational study
Source: Front Pharmacol. 2023 Apr 6;14:1135736. doi: 10.3389/fphar.2023.1135736 (PMC10116053; doi:10.3389/fphar.2023.1135736)
Supplement: Supplementary file 1 [file DataSheet1.docx]

**Table 1. Social-demographic variables in association with folic acid use among pregnant women (N = 2803)**

| Variables | Non-users  n=1207 | Users  n=1596 | χ2 | p value |
| --- | --- | --- | --- | --- |
| Age |  |  | 0.090 | 0.764 |
| <35 years | 1006 (83.3) | 1337 (83.8) |  |  |
| ≥35 years | 201 (16.7) | 259 (16.2) |  |  |
| Ethnicity |  |  | 0.440 | 0.507 |
| Han Chinese | 1180 (97.8) | 1566 (98.1) |  |  |
| Ethnic minority | 27 ( 2.2) | 30 ( 1.9) |  |  |
| Employment^#^ |  |  | **6.380** | **0.012** |
| Employed | 987 (81.8) | 1355 (85.3) |  |  |
| Unemployed/housewife | 220 (18.2) | 233 (14.7) |  |  |
| Education level^#^ |  |  | **12.545** | **0.014** |
| Primary education | 69 ( 5.7) | 76 ( 4.8) |  |  |
| Secondary education | 117 ( 9.7) | 117 ( 7.4) |  |  |
| College | 305 (25.3) | 360 (22.7) |  |  |
| Bachelor | 542 (44.9) | 757 (47.7) |  |  |
| Master’s and above | 174 (14.4) | 277 (17.5) |  |  |
| Marital status |  |  | 0.058 | 0.810 |
| Single/divorced | 56 ( 4.6) | 71 ( 4.4) |  |  |
| Married | 1151 (95.4) | 1525 (95.6) |  |  |
| Local residence |  |  | 0.997 | 0.318 |
| Shanghai | 351 (29.1) | 492 (30.8) |  |  |
| Non-Shanghai | 856 (70.9) | 1104 (69.2) |  |  |
| Medical insurance |  |  | 0.108 | 0.742 |
| No medical insurance | 154 (12.8) | 197 (12.3) |  |  |
| Have medical insurance | 1053 (87.2) | 1399 (87.7) |  |  |
| Social support |  |  | 5.872 | 0.053 |
| Low | 457 (37.9) | 579 (36.3) |  |  |
| Medium | 407 (33.7) | 494 (31.0) |  |  |
| High | 343 (28.4) | 523 (32.8) |  |  |

^#^missing data.

**Table 2. Obstetric variables in association with folic acid use among pregnant women (N = 2803)**

| Variables | Non-users  n=1207 | Users  n=1596 | χ2 | p value |
| --- | --- | --- | --- | --- |
| Gestational age |  |  | **740.362** | **<0.001** |
| First trimester | 63 ( 5.2) | 852 (53.4) |  |  |
| Second trimester | 555 (46.0) | 427 (26.8) |  |  |
| Third trimester | 589 (48.8) | 317 (19.9) |  |  |
| PPBMI |  |  | 0.894 | 0.827 |
| Underweight | 144 (11.9) | 176 (11.0) |  |  |
| Normal weight | 864 (71.6) | 1150 (72.1) |  |  |
| Overweight | 171 (14.2) | 227 (14.2) |  |  |
| Obese | 28 ( 2.3) | 43 ( 2.7) |  |  |
| Conception method |  |  | 0.039 | 0.843 |
| Natural conception | 1110 (92.0) | 1471 (92.2) |  |  |
| Artificial conception | 97 ( 8.0) | 125 ( 7.8) |  |  |
| Parity |  |  | 0.072 | 0.788 |
| Primipara | 909 (75.3) | 1209 (75.8) |  |  |
| Multipara | 298 (24.7) | 387 (24.2) |  |  |
| History of adverse pregnancy |  |  | 0.033 | 0.856 |
| No | 1063 (88.1) | 1402 (87.8) |  |  |
| Yes | 144 (11.9) | 194 (12.2) |  |  |
| Embryo number |  |  | 3.152 | 0.076 |
| Singleton | 1192 (98.8) | 1562 (97.9) |  |  |
| Twin | 15 ( 1.2) | 34 ( 2.1) |  |  |
| Pregnancy risk grading |  |  | 0.673 | 0.714 |
| Green | 508 (42.1) | 665 (41.7) |  |  |
| Yellow | 633 (52.4) | 832 (52.1) |  |  |
| Orange | 66 ( 5.5) | 99 ( 6.2) |  |  |

PPBMI: pre-pregnancy body mass index.

**Table 3. Social-demographic variables in association with calcium use among pregnant women (N = 2803)**

| Variables | Non-users  n=1167 | Users  n=1636 | χ2 | p value |
| --- | --- | --- | --- | --- |
| Age |  |  | 0.776 | 0.378 |
| <35 years | 984 (84.3) | 1359 (83.1) |  |  |
| ≥35 years | 183 (15.7) | 277 (16.9) |  |  |
| Ethnicity |  |  | 0.550 | 0.458 |
| Han Chinese | 1146 (98.2) | 1600 (97.8) |  |  |
| Ethnic minority | 21 ( 1.8) | 36 ( 2.2) |  |  |
| Employment^#^ |  |  | **7.243** | **0.007** |
| Employed | 952 (81.6) | 1390 (85.4) |  |  |
| Unemployed/housewife | 215 (18.4) | 238 (14.6) |  |  |
| Education level^#^ |  |  | **15.829** | **0.003** |
| Primary education | 81 ( 6.9) | 64 ( 3.9) |  |  |
| Secondary education | 99 ( 8.5) | 135 ( 8.3) |  |  |
| College | 290 (24.9) | 375 (23.0) |  |  |
| Bachelor | 513 (44.0) | 786 (48.3) |  |  |
| Master’s and above | 183 (15.7) | 268 (16.5) |  |  |
| Marital status |  |  | **4.990** | **0.025** |
| Single/divorced | 65 ( 5.6) | 62 ( 3.8) |  |  |
| Married | 1102 (94.4) | 1574 (96.2) |  |  |
| Local residence |  |  | 0.253 | 0.615 |
| Shanghai | 357 (30.6) | 486 (29.7) |  |  |
| Non-Shanghai | 810 (69.4) | 1150 (70.3) |  |  |
| Medical insurance |  |  | **8.966** | **0.003** |
| No medical insurance | 172 (14.7) | 179 (10.9) |  |  |
| Have medical insurance | 995 (85.3) | 1457 (89.1) |  |  |
| Social support |  |  | 1.036 | 0.596 |
| Low | 428 (36.7) | 608 (37.2) |  |  |
| Medium | 387 (33.2) | 514 (31.4) |  |  |
| High | 352 (30.2) | 514 (31.4) |  |  |

^#^missing data.

**Table 4. Obstetric variables in association with calcium use among pregnant women (N = 2803)**

| Variables | Non-users  n=1167 | Users  n=1636 | χ2 | p value |
| --- | --- | --- | --- | --- |
| Gestational age |  |  | **942.451** | **<0.001** |
| First trimester | 756 (64.8) | 159 ( 9.7) |  |  |
| Second trimester | 233 (20.0) | 749 (45.8) |  |  |
| Third trimester | 178 (15.3) | 728 (44.5) |  |  |
| PPBMI |  |  | 3.295 | 0.348 |
| Underweight | 132 (11.3) | 188 (11.5) |  |  |
| Normal weight | 833 (71.4) | 1181 (72.2) |  |  |
| Overweight | 165 (14.1) | 233 (14.2) |  |  |
| Obese | 37 ( 3.2) | 34 ( 2.1) |  |  |
| Conception method |  |  | 0.007 | 0.935 |
| Natural conception | 1074 (92.0) | 1507 (92.1) |  |  |
| Artificial conception | 93 ( 8.0) | 129 ( 7.9) |  |  |
| Parity |  |  | 0.485 | 0.486 |
| Primipara | 874 (74.9) | 1244 (76.0) |  |  |
| Multipara | 293 (25.1) | 392 (24.0) |  |  |
| History of adverse pregnancy |  |  | 1.053 | 0.305 |
| No | 1035 (88.7) | 1430 (87.4) |  |  |
| Yes | 132 (11.3) | 206 (12.6) |  |  |
| Embryo number |  |  | 2.680 | 0.102 |
| Singleton | 1141 (97.8) | 1613 (98.6) |  |  |
| Twin | 26 ( 2.2) | 23 ( 1.4) |  |  |
| Pregnancy risk grading |  |  | 0.846 | 0.655 |
| Green | 490 (42.0) | 683 (41.7) |  |  |
| Yellow | 603 (51.7) | 862 (52.7) |  |  |
| Orange | 74 ( 6.3) | 91 ( 5.6) |  |  |

PPBMI: pre-pregnancy body mass index.

**Table 5. Social-demographic variables in association with iron use among pregnant women (N = 2803)**

| Variables | Non-users  n=1940 | Users  n=863 | χ2 | p value |
| --- | --- | --- | --- | --- |
| Age |  |  | 0.386 | 0.534 |
| <35 years | 1616 (83.3) | 727 (84.2) |  |  |
| ≥35 years | 324 (16.7) | 136 (15.8) |  |  |
| Ethnicity |  |  | 1.739 | 0.187 |
| Han Chinese | 1896 (97.7) | 850 (98.5) |  |  |
| Ethnic minority | 44 ( 2.3) | 13 ( 1.5) |  |  |
| Employment^#^ |  |  | **11.077** | **0.001** |
| Employed | 1594 (82.2) | 748 (87.3) |  |  |
| Unemployed/housewife | 344 (17.8) | 109 (12.7) |  |  |
| Education level^#^ |  |  | **26.730** | **<0.001** |
| Primary education | 121 ( 6.2) | 24 ( 2.8) |  |  |
| Secondary education | 170 ( 8.8) | 64 ( 7.5) |  |  |
| College | 481 (24.8) | 184 (21.5) |  |  |
| Bachelor | 879 (45.4) | 420 (49.0) |  |  |
| Master’s and above | 286 (14.8) | 165 (19.3) |  |  |
| Marital status |  |  | 1.007 | 0.316 |
| Single/divorced | 93 ( 4.8) | 34 ( 3.9) |  |  |
| Married | 1847 (95.2) | 829 (96.1) |  |  |
| Local residence |  |  | 0.726 | 0.394 |
| Shanghai | 593 (30.6) | 250 (29.0) |  |  |
| Non-Shanghai | 1347 (69.4) | 613 (71.0) |  |  |
| Medical insurance |  |  | **7.443** | **0.006** |
| No medical insurance | 265 (13.7) | 86 (10.0) |  |  |
| Have medical insurance | 1675 (86.3) | 777 (90.0) |  |  |
| Social support |  |  | 2.940 | 0.230 |
| Low | 733 (37.8) | 303 (35.1) |  |  |
| Medium | 625 (32.2) | 276 (32.0) |  |  |
| High | 582 (30.0) | 284 (32.9) |  |  |

^#^missing data.

**Table 6. Obstetric variables in association with iron use among pregnant women (N = 2803)**

| Variables | Non-users  n=1940 | Users  n=863 | χ2 | p value |
| --- | --- | --- | --- | --- |
| Gestational age |  |  | **655.463** | **<0.001** |
| First trimester | 843 (43.5) | 72 ( 8.3) |  |  |
| Second trimester | 750 (38.7) | 232 (26.9) |  |  |
| Third trimester | 347 (17.9) | 559 (64.8) |  |  |
| PPBMI |  |  | **18.255** | **<0.001** |
| Underweight | 205 (10.6) | 115 (13.3) |  |  |
| Normal weight | 1376 (70.9) | 638 (73.9) |  |  |
| Overweight | 300 (15.5) | 98 (11.4) |  |  |
| Obese | 59 ( 3.0) | 12 ( 1.4) |  |  |
| Conception method |  |  | **5.409** | **0.020** |
| Natural conception | 1771 (91.3) | 810 (93.9) |  |  |
| Artificial conception | 169 ( 8.7) | 53 ( 6.1) |  |  |
| Parity |  |  | 0.751 | 0.386 |
| Primipara | 1475 (76.0) | 643 (74.5) |  |  |
| Multipara | 465 (24.0) | 220 (25.5) |  |  |
| History of adverse pregnancy |  |  | **10.726** | **0.001** |
| No | 1680 (86.6) | 785 (91.0) |  |  |
| Yes | 260 (13.4) | 78 ( 9.0) |  |  |
| Embryo number |  |  | 3.611 | 0.057 |
| Singleton | 1900 (97.9) | 854 (99.0) |  |  |
| Twin | 40 ( 2.1) | 9 ( 1.0) |  |  |
| Pregnancy risk grading |  |  | **6.131** | **0.047** |
| Green | 785 (40.5) | 388 (45.0) |  |  |
| Yellow | 1032 (53.2) | 433 (50.2) |  |  |
| Orange | 123 ( 6.3) | 42 ( 4.9) |  |  |

PPBMI: pre-pregnancy body mass index.

**Table 7. Social-demographic variables in association with iodine use among pregnant women (N = 2803)**

| Variables | Non-users  n=2762 | Users  n=41 | χ2 | p value |
| --- | --- | --- | --- | --- |
| Age |  |  | 0.539 | 0.463 |
| <35 years | 2307 (83.5) | 36 (87.8) |  |  |
| ≥35 years | 455 (16.5) | 5 (12.2) |  |  |
| Ethnicity |  |  | 0.034 | 0.853 |
| Han Chinese | 2706 (98.0) | 40 (97.6) |  |  |
| Ethnic minority | 56 ( 2.0) | 1 ( 2.4) |  |  |
| Employment^#^ |  |  | 0.335 | 0.563 |
| Employed | 2309 (83.8) | 33 (80.5) |  |  |
| Unemployed/housewife | 445 (16.2) | 8 (19.5) |  |  |
| Education level^#^ |  |  | 7.060 | 0.133 |
| Primary education | 145 ( 5.3) | 0 ( 0.0) |  |  |
| Secondary education | 233 ( 8.5) | 1 ( 2.5) |  |  |
| College | 656 (23.8) | 9 (22.5) |  |  |
| Bachelor | 1280 (46.5) | 19 (47.5) |  |  |
| Master’s and above | 440 (16.0) | 11 (27.5) |  |  |
| Marital status |  |  | 0.012 | 0.709 |
| Single/divorced | 125 ( 4.5) | 2 ( 4.9) |  |  |
| Married | 2637 (95.5) | 39 (95.1) |  |  |
| Local residence |  |  | 3.783 | 0.052 |
| Shanghai | 825 (29.9) | 18 (43.9) |  |  |
| Non-Shanghai | 1937 (70.1) | 23 (56.1) |  |  |
| Medical insurance |  |  | **3.862** | **0.049** |
| No medical insurance | 350 (12.7) | 1 ( 2.4) |  |  |
| Have medical insurance | 2412 (87.3) | 40 (97.6) |  |  |
| Social support |  |  | 2.289 | 0.318 |
| Low | 1025 (37.1) | 11 (26.8) |  |  |
| Medium | 884 (32.0) | 17 (41.5) |  |  |
| High | 853 (30.9) | 13 (31.7) |  |  |

^#^missing data.

**Table 8. Obstetric variables in association with iodine use among pregnant women (N = 2803)**

| Variables | Non-users  n=2762 | Users  n=41 | χ2 | p value |
| --- | --- | --- | --- | --- |
| Gestational age |  |  | 0.693 | 0.707 |
| First trimester | 904 (32.7) | 11 (26.8) |  |  |
| Second trimester | 967 (35.0) | 15 (36.6) |  |  |
| Third trimester | 891 (32.3) | 15 (36.6) |  |  |
| PPBMI |  |  | 1.877 | 0.598 |
| Underweight | 315 (11.4) | 5 (12.2) |  |  |
| Normal weight | 1982 (71.8) | 32 (78.0) |  |  |
| Overweight | 394 (14.3) | 4 ( 9.8) |  |  |
| Obese | 71 ( 2.6) | 0 ( 0.0) |  |  |
| Conception method |  |  | 1.714 | 0.190 |
| Natural conception | 2541 (92.0) | 40 (97.6) |  |  |
| Artificial conception | 221 ( 8.0) | 1 ( 2.4) |  |  |
| Parity |  |  | 3.377 | 0.066 |
| Primipara | 2082 (75.4) | 36 (87.8) |  |  |
| Multipara | 680 (24.6) | 5 (12.2) |  |  |
| History of adverse pregnancy |  |  | 0.001 | 0.978 |
| No | 2429 (87.9) | 36 (87.8) |  |  |
| Yes | 333 (12.1) | 5 (12.2) |  |  |
| Embryo number |  |  | 2.373 | 0.123 |
| Singleton | 2715 (98.3) | 39 (95.1) |  |  |
| Twin | 47 ( 1.7) | 2 ( 4.9) |  |  |
| Pregnancy risk grading |  |  | 1.947 | 0.378 |
| Green | 1152 (41.7) | 21 (51.2) |  |  |
| Yellow | 1448 (52.4) | 17 (41.5) |  |  |
| Orange | 162 ( 5.9) | 3 ( 7.3) |  |  |

PPBMI: pre-pregnancy body mass index.

**Table 9. Social-demographic variables in association with zinc use among pregnant women (N = 2803)**

| Variables | Non-users  n=2716 | Users  n=87 | χ2 | p value |
| --- | --- | --- | --- | --- |
| Age |  |  | 0.045 | 0.832 |
| <35 years | 2271 (83.6) | 72 (82.8) |  |  |
| ≥35 years | 445 (16.4) | 15 (17.2) |  |  |
| Ethnicity |  |  | 0.902 | 0.342 |
| Han Chinese | 2662 (98.0) | 84 (96.6) |  |  |
| Ethnic minority | 54 ( 2.0) | 3 ( 3.4) |  |  |
| Employment^#^ |  |  | 0.071 | 0.790 |
| Employed | 2270 (83.8) | 72 (82.8) |  |  |
| Unemployed/housewife | 438 (16.2) | 15 (17.2) |  |  |
| Education level^#^ |  |  | 1.825 | 0.768 |
| Primary education | 143 ( 5.3) | 2 ( 2.3) |  |  |
| Secondary education | 227 ( 8.4) | 7 ( 8.0) |  |  |
| College | 645 (23.8) | 20 (23.0) |  |  |
| Bachelor | 1257 (46.4) | 42 (48.3) |  |  |
| Master’s and above | 435 (16.1) | 16 (18.4) |  |  |
| Marital status |  |  | 0.307 | 0.579 |
| Single/divorced | 122 ( 4.5) | 5 ( 5.7) |  |  |
| Married | 2594 (95.5) | 82 (94.3) |  |  |
| Local residence |  |  | 0.264 | 0.607 |
| Shanghai | 819 (30.2) | 24 (27.6) |  |  |
| Non-Shanghai | 1897 (69.8) | 63 (72.4) |  |  |
| Medical insurance |  |  | 0.907 | 0.341 |
| No medical insurance | 343 (12.6) | 8 ( 9.2) |  |  |
| Have medical insurance | 2373 (87.4) | 79 (90.8) |  |  |
| Social support |  |  | 1.990 | 0.370 |
| Low | 1010 (37.2) | 26 (29.9) |  |  |
| Medium | 869 (32.0) | 32 (36.8) |  |  |
| High | 837 (30.8) | 29 (33.3) |  |  |

^#^missing data.

**Table 10. Obstetric variables in association with** **zinc use among pregnant women (N = 2803)**

| Variables | Non-users  n=2716 | Users  n=87 | χ2 | p value |
| --- | --- | --- | --- | --- |
| Gestational age |  |  | 5.922 | 0.052 |
| First trimester | 897 (33.0) | 18 (20.7) |  |  |
| Second trimester | 946 (34.8) | 36 (41.4) |  |  |
| Third trimester | 873 (32.1) | 33 (37.9) |  |  |
| PPBMI |  |  | 4.915 | 0.178 |
| Underweight | 306 (11.3) | 14 (16.1) |  |  |
| Normal weight | 1956 (72.0) | 58 (66.7) |  |  |
| Overweight | 383 (14.1) | 15 (17.2) |  |  |
| Obese | 71 ( 2.6) | 0 ( 0.0) |  |  |
| Conception method |  |  | 1.359 | 0.244 |
| Natural conception | 2498 (92.0) | 83 (95.4) |  |  |
| Artificial conception | 218 ( 8.0) | 4 ( 4.6) |  |  |
| Parity |  |  | 0.482 | 0.488 |
| Primipara | 2055 (75.7) | 63 (72.4) |  |  |
| Multipara | 661 (24.3) | 24 (27.6) |  |  |
| History of adverse pregnancy |  |  | 0.249 | 0.618 |
| No | 2387 (87.9) | 78 (89.7) |  |  |
| Yes | 329 (12.1) | 9 (10.3) |  |  |
| Embryo number |  |  | 0.187 | 0.665 |
| Singleton | 2668 (98.2) | 86 (98.9) |  |  |
| Twin | 48 ( 1.8) | 1 ( 1.1) |  |  |
| Pregnancy risk grading |  |  | 0.018 | 0.991 |
| Green | 1136 (41.8) | 37 (42.5) |  |  |
| Yellow | 1420 (52.3) | 45 (51.7) |  |  |
| Orange | 160 ( 5.9) | 5 ( 5.7) |  |  |

PPBMI: pre-pregnancy body mass index.

**Table 11. Social-demographic variables in association with vitamins use among pregnant women (N = 2803)**

| Variables | Non-users  n=1725 | Users  n=1078 | χ2 | p value |
| --- | --- | --- | --- | --- |
| Age |  |  | **7.479** | **0.006** |
| <35 years | 1468 (85.1) | 875 (81.2) |  |  |
| ≥35 years | 257 (14.9) | 203 (18.8) |  |  |
| Ethnicity |  |  | **4.938** | **0.026** |
| Han Chinese | 1698 (98.4) | 1048 (97.2) |  |  |
| Ethnic minority | 27 ( 1.6) | 30 ( 2.8) |  |  |
| Employment^#^ |  |  | **7.476** | **0.006** |
| Employed | 1417 (82.3) | 925 (86.2) |  |  |
| Unemployed/housewife | 305 (17.7) | 148 (13.8) |  |  |
| Education level^#^ |  |  | **46.312** | **<0.001** |
| Primary education | 108 ( 6.3) | 37 ( 3.4) |  |  |
| Secondary education | 158 ( 9.2) | 76 ( 7.1) |  |  |
| College | 447 (26.0) | 218 (20.3) |  |  |
| Bachelor | 779 (45.3) | 520 (48.5) |  |  |
| Master’s and above | 229 (13.3) | 222 (20.7) |  |  |
| Marital status |  |  | 1.190 | 0.275 |
| Single/divorced | 84 ( 4.9) | 43 ( 4.0) |  |  |
| Married | 1641 (95.1) | 1035 (96.0) |  |  |
| Local residence |  |  | **19.977** | **<0.001** |
| Shanghai | 466 (27.0) | 377 (35.0) |  |  |
| Non-Shanghai | 1259 (73.0) | 701 (65.0) |  |  |
| Medical insurance |  |  | **11.565** | **0.001** |
| No medical insurance | 245 (14.2) | 106 ( 9.8) |  |  |
| Have medical insurance | 1480 (85.8) | 972 (90.2) |  |  |
| Social support |  |  | **7.474** | **0.024** |
| Low | 666 (38.6) | 370 (34.3) |  |  |
| Medium | 557 (32.3) | 344 (31.9) |  |  |
| High | 502 (29.1) | 364 (33.8) |  |  |

^#^missing data.

**Table 12. Obstetric variables in association with** **vitamins use among pregnant women (N = 2803)**

| Variables | Non-users  n=1725 | Users  n=1078 | χ2 | p value |
| --- | --- | --- | --- | --- |
| Gestational age |  |  | **34.428** | **<0.001** |
| First trimester | 632 (36.6) | 283 (26.3) |  |  |
| Second trimester | 584 (33.9) | 398 (36.9) |  |  |
| Third trimester | 509 (29.5) | 397 (36.8) |  |  |
| PPBMI |  |  | 5.616 | 0.132 |
| Underweight | 204 (11.8) | 116 (10.8) |  |  |
| Normal weight | 1238 (71.8) | 776 (72.0) |  |  |
| Overweight | 232 (13.4) | 166 (15.4) |  |  |
| Obese | 51 ( 3.0) | 20 ( 1.9) |  |  |
| Conception method |  |  | 0.653 | 0.419 |
| Natural conception | 1594 (92.4) | 987 (91.6) |  |  |
| Artificial conception | 131 ( 7.6) | 91 ( 8.4) |  |  |
| Parity |  |  | 3.086 | 0.079 |
| Primipara | 1284 (74.4) | 834 (77.4) |  |  |
| Multipara | 441 (25.6) | 244 (22.6) |  |  |
| History of adverse pregnancy |  |  | 0.228 | 0.633 |
| No | 1521 (88.2) | 944 (87.6) |  |  |
| Yes | 204 (11.8) | 134 (12.4) |  |  |
| Embryo number |  |  | 3.325 | 0.068 |
| Singleton | 1701 (98.6) | 1053 (97.7) |  |  |
| Twin | 24 ( 1.4) | 25 ( 2.3) |  |  |
| Pregnancy risk grading |  |  | **9.453** | **0.009** |
| Green | 734 (42.6) | 439 (40.7) |  |  |
| Yellow | 908 (52.6) | 557 (51.7) |  |  |
| Orange | 83 ( 4.8) | 82 ( 7.6) |  |  |

PPBMI: pre-pregnancy body mass index.

**Table 13. Social-demographic variables in association with DHA use among pregnant women (N = 2803)**

| Variables | Non-users  n=2056 | Users  n=747 | χ2 | p value |
| --- | --- | --- | --- | --- |
| Age |  |  | **4.509** | **0.034** |
| <35 years | 1737 (84.5) | 606 (81.1) |  |  |
| ≥35 years | 319 (15.5) | 141 (18.9) |  |  |
| Ethnicity |  |  | 0.723 | 0.395 |
| Han Chinese | 2017 (98.1) | 729 (97.6) |  |  |
| Ethnic minority | 39 ( 1.9) | 18 ( 2.4) |  |  |
| Employment^#^ |  |  | **29.268** | **<0.001** |
| Employed | 1672 (81.5) | 670 (90.1) |  |  |
| Unemployed/housewife | 379 (18.5) | 74 ( 9.9) |  |  |
| Education level^#^ |  |  | **31.757** | **<0.001** |
| Primary education | 129 ( 6.3) | 16 ( 2.2) |  |  |
| Secondary education | 176 ( 8.6) | 58 ( 7.8) |  |  |
| College | 493 (24.0) | 172 (23.1) |  |  |
| Bachelor | 956 (46.6) | 343 (46.2) |  |  |
| Master’s and above | 297 (14.5) | 154 (20.7) |  |  |
| Marital status |  |  | 3.301 | 0.069 |
| Single/divorced | 102 ( 5.0) | 25 ( 3.3) |  |  |
| Married | 1954 (95.0) | 722 (96.7) |  |  |
| Local residence |  |  | **10.839** | **0.001** |
| Shanghai | 583 (28.4) | 260 (34.8) |  |  |
| Non-Shanghai | 1473 (71.6) | 487 (65.2) |  |  |
| Medical insurance |  |  | **24.749** | **<0.001** |
| No medical insurance | 296 (14.4) | 55 ( 7.4) |  |  |
| Have medical insurance | 1760 (85.6) | 692 (92.6) |  |  |
| Social support |  |  | 1.732 | 0.421 |
| Low | 774 (37.6) | 262 (35.1) |  |  |
| Medium | 660 (32.1) | 241 (32.3) |  |  |
| High | 622 (30.3) | 244 (32.7) |  |  |

^#^missing data.

**Table 14. Obstetric variables in association with** **DHA use among pregnant women (N = 2803)**

| Variables | Non-users  n=2056 | Users  n=747 | χ2 | p value |
| --- | --- | --- | --- | --- |
| Gestational age |  |  | **152.287** | **<0.001** |
| First trimester | 806 (39.2) | 109 (14.6) |  |  |
| Second trimester | 640 (31.1) | 342 (45.8) |  |  |
| Third trimester | 610 (29.7) | 296 (39.6) |  |  |
| PPBMI |  |  | 4.091 | 0.252 |
| Underweight | 242 (11.8) | 78 (10.4) |  |  |
| Normal weight | 1471 (71.5) | 543 (72.7) |  |  |
| Overweight | 285 (13.9) | 113 (15.1) |  |  |
| Obese | 58 ( 2.8) | 13 ( 1.7) |  |  |
| Conception method |  |  | 0.084 | 0.771 |
| Natural conception | 1895 (92.2) | 686 (91.8) |  |  |
| Artificial conception | 161 ( 7.8) | 61 ( 8.2) |  |  |
| Parity |  |  | 0.424 | 0.515 |
| Primipara | 1547 (75.2) | 571 (76.4) |  |  |
| Multipara | 509 (24.8) | 176 (23.6) |  |  |
| History of adverse pregnancy |  |  | 0.636 | 0.425 |
| No | 1802 (87.6) | 663 (88.8) |  |  |
| Yes | 254 (12.4) | 84 (11.2) |  |  |
| Embryo number |  |  | 0.450 | 0.502 |
| Singleton | 2018 (98.2) | 736 (98.5) |  |  |
| Twin | 38 ( 1.8) | 11 ( 1.5) |  |  |
| Pregnancy risk grading |  |  | **18.121** | **<0.001** |
| Green | 886 (43.1) | 287 (38.4) |  |  |
| Yellow | 1032 (50.2) | 433 (58.0) |  |  |
| Orange | 138 ( 6.7) | 27 ( 3.6) |  |  |

PPBMI: pre-pregnancy body mass index.
